# Research can be integrated into public health policy-making: global lessons for and from Spanish economic evaluations

**DOI:** 10.1186/s12961-022-00875-6

**Published:** 2022-06-18

**Authors:** Marta Trapero-Bertran, Subhash Pokhrel, Stephen Hanney

**Affiliations:** 1 Basic Sciences Department, Patients Institute, Universitat Internacional de Catalunya, Barcelona, Spain; 2 Health Economics Research Group, Department of Health Sciences, Brunel University London, London, UK

**Keywords:** Public health, Policy-making, Economic evaluation, Knowledge translation, Research impact, Sustainable Development Goals, Tobacco control, COVID-19, Horizon Europe, Return on investment

## Abstract

WHO promotes the use of research in policy-making to drive improvements in health, including in achieving Sustainable Development Goals such as tobacco control. The European Union’s new €95 billion Horizon Europe research framework programme parallels these aims, and also includes commitments to fund economic evaluations. However, researchers often express frustration at the perceived lack of attention to scientific evidence during policy-making. For example, some researchers claim that evidence regarding the return on investment from optimal implementation of evidence-based policies is frequently overlooked. An increasingly large body of literature acknowledges inevitable barriers to research use, but also analyses facilitators encouraging such use. This opinion piece describes how some research is integrated into policy-making. It highlights two recent reviews. One examines impact assessments of 36 multi-project research programmes and identifies three characteristics of projects more likely to influence policy-making. These include a focus on healthcare system needs, engagement of stakeholders, and research conducted for organizations supported by structures to receive and use evidence. The second review suggests that such characteristics are likely to occur as part of a comprehensive national health research system strategy, especially one integrated into the healthcare system. We also describe two policy-informing economic evaluations conducted in Spain. These examined the most cost-effective package of evidence-based tobacco control interventions and the cost-effectiveness of different strategies to increase screening coverage for cervical cancer. Both projects focused on issues of healthcare concern and involved considerable stakeholder engagement. The Spanish examples reinforce some lessons from the global literature and, therefore, could help demonstrate to authorities in Spain the value of developing comprehensive health research systems, possibly following the interfaces and receptor model. The aim of this would be to integrate needs assessment and stakeholder engagement with structures spanning the research and health systems. In such structures, economic evaluation evidence could be collated, analysed by experts in relation to healthcare needs, and fed into both policy-making as appropriate, and future research calls. The increasingly large local and global evidence base on research utilization could inform detailed implementation of this approach once accepted as politically desirable. Given the COVID-19 pandemic, increasing the cost-effectiveness of healthcare systems and return on investment of public health interventions becomes even more important.

## Background

WHO emphasizes the importance of the use of research in policy-making to drive improvements in health services and population health and well-being [1]. Similarly, the European Union (EU)’s new €95 billion Horizon Europe Research and Innovation Framework Programme, which runs from 2021 to 2027, aims to strengthen the impact of research and innovation on policies and “to address global challenges, including climate change and the Sustainable Development Goals [SDGs]” [2].

Health issues are important in various SDGs, with SDG 3 explicitly proclaiming the need to “ensure healthy lives and promote well-being for all at all ages” [3]. Many of the specific items within SDG 3 cover the public health problems that are responsible for a high burden of disease and substantial costs to health systems. For example, SDG 3a states, “Strengthen the implementation of the WHO Framework Convention on Tobacco Control in all countries, as appropriate.”. In describing its plans for health research in Horizon Europe, the European Commission set out how it would address a series of complex and interdependent challenges, “as part of the Union’s commitment to global health, universal health coverage and SDG 3” [2]. The document immediately went on to claim that more than 790,000 deaths annually in Europe were due to areas of public health concern such as smoking, drinking, physical inactivity and obesity, and it linked various of its targets to specific detailed SDG 3 targets.

Other aims of the Horizon Europe plan include contributing to Europe’s Beating Cancer Plan to support EU member states in improving cancer control and care. Within the broad commitment to use research and innovation to ensure access to innovative, sustainable and high-quality healthcare, there is a commitment to support “innovative full health technology assessment methods (i.e. including all relevant aspects such as clinical effectiveness, cost-effectiveness, ethics, organizational aspects, etc.) to support better allocation of resources” [2]*.* There is also a commitment to work with health and social care organizations to boost research for policy-making: “Specific objectives would be to provide evidence for innovative solutions that support cost-effective and fiscally sustainable health and care policies” [2].

Therefore, there is widespread commitment to using research, including economic evaluations, in health policy-making. In practice, however, it has often seemed difficult to achieve this, and thought is given as to how best to address the challenges.

The aim of this opinion paper is to propose ways in which economic evaluations could be more fully integrated into health policy-making in Spain and its autonomous regions. Therefore, this background section continues with a brief overview of, first, the international debate about the use of evidence in health policy-making, and second, the current state of health policy-making in Spain and the use of economic evaluations. The main section then extracts lessons from the global literature by drawing in a more detailed way on two recent evidence syntheses conducted by team members that use different perspectives to those traditionally adopted to collect and analyse data on the use of research evidence in policy-making. The main section reinforces the lessons with new analysis of two examples of economic evaluations in Spain, on tobacco control and cancer screening, undertaken by team members that did have some influence on health policy-making. Finally, this material is used to make proposals as to how evaluations could be more fully integrated into Spanish health policy-making.

### International debate about evidence use in policy-making

Despite considerable efforts, researchers and others often report frustrations with what they perceive to be a lack of attention to scientific evidence when policies are made. The *World health report 2013* claimed that, “because many existing cost-effective interventions are not widely used, there is a particular need to close the gap between existing knowledge and action” [1]. Health technology assessments (HTAs) evaluate the benefits and costs of health technologies, and when used can inform policy decisions about whether a particular intervention represents value for money. In Bangladesh, India and Viet Nam [4], and in low- and middle-income countries (LMICs) in general [5, 6], it is noted that economic evaluations and HTAs seem to make only a limited impact on health policy decisions, and less, it is claimed, than in certain high-income countries. Nevertheless, even from North American perspectives, it is also suggested there are limitations on the impact economic evaluations seem to make on health policy decisions, even when they might be taken into consideration [7]. A recent systematic review of “economic evaluations of public health implementation-interventions” [8] also highlighted the need for greater application and use of economic evaluation to understand the cost-effectiveness of alternative implementation efforts in order to inform public health policy and investment decisions.

Therefore, the issues around the claims of underuse of research in health policy-making are complex. For example, partly the problem is that suboptimal use appears to be made of existing research on new interventions. A further issue is that the need for research and information on the value for money or return on investment (ROI) from optimal implementation of evidence-based policies has been widely overlooked.

The claims about the sometimes limited use of research evidence are not restricted to economic evaluations, but go across the spectrum of health research, and the full spectrum of high-, middle- and low-income countries [9–11]. For example, in 2016, WHO’s Regional Office for Europe stated: “Health policy, however, is often not optimally informed by this available evidence” [9]. The *World health report 2013* also identified diverse challenges facing attempts to increase the use of health research in policy-making, and discussed how it was vital to recognize the needs and perspectives of policy-makers [1].

There have been important attempts to analyse both the complexities around using evidence in inevitably politicized processes [12], and the frustrations of limitations on the use of research within the context of the realities of policy-making and the many influences that invariably impinge on it [13]. In this latter analysis, Cairney and Oliver also suggested that researchers need to recognize several key points. First, the other factors that influence policy-makers include their own beliefs and the views of interest groups, and second, researchers might have to decide how far they wished to go in trying to persuade policy-makers to act. Perhaps along somewhat similar lines, Greenhalgh and Fahy in 2018 suggested that the use of research was characterized by factors such an ethical commitment by researchers [14].

At a local policy-making level within a healthcare system, the use of research evidence can often be complex, as illustrated by the increasing range of terms adopted to best account for this. Such terms include knowledge translation, knowledge mobilization and knowledge transformation, in which it is acknowledged that local healthcare policy-makers transform research evidence as they consider and incorporate it alongside other sources of evidence [15].

Oliver et al. (2014) reviewed 145 studies of factors affecting the use of evidence in policy, mostly in the health field, and found that collaboration between researchers and policy-makers was one of the most frequently reported facilitators [16]. A recent historical analysis identified a variety of ways of promoting collaboration between researchers and research users [17]. Particular attention has focused on the best ways of undertaking stakeholder engagement to increase health research use [18, 19].

While, as noted above, the actions of individual researchers can be important, the details of many interventions to increase policy-makers’ capacity to use research were also analysed in a realist scoping review, with the importance of genuine collaboration and system supports highlighted [20]. Yet another recent review identified 64 organizational factors (placed into five main categories with 18 subcategories) that advanced research use in public health policy-making, and included a call for frameworks that combine different approaches [21].

A stream of analysis in the United Kingdom going back almost 40 years to Kogan and Henkel (and colleagues) [22–24], and also in Canada to the work of Lomas (and others) [25], emphasized the importance of a collaborative approach between researchers conducting policy-relevant studies and the intended policy users of such research. Part of this analysis in the United Kingdom led to the 2002 “interfaces and receptor” model developed in a report to WHO on research use in health policy-making [23]. It was subsequently described, in Spanish, in *Medicina Clinica* [26]. This model captured the dual focus on both the interaction between researchers and users (especially around the needs of the healthcare system) and the importance of structures in the form of receptor bodies organized to receive and use the findings. The stream of analysis showed that the relationship between research and policy-making would vary according to a range of factors such as the type of research and the type of decisions involved. In particular, it became clear, as elaborated below, that when drawing on economic evaluations, there were various technical considerations that might be best considered by specialist advisory bodies [23]. This line of thinking was also informed by a European working group on HTAs that had identified the importance of the institutional arrangements in each particular country for determining the level of evidence use [27].

Finally, the need for evidence-based insights to inform government responses is recognized as being more acute during the global COVID-19 pandemic, with the important role of knowledge translation platforms being emphasized [28, 29].

### Use of economic evaluations in health policy-making in Spain and its autonomous regions

While the ever-expanding global evidence base on research utilization is useful, the determinants of evidence use in public health policy-making can differ between country and policy context, as again highlighted by a recent study across six EU nations [30]. In Spain, although Article 31.2 of the Spanish Constitution of 1978 includes the principles of equity and efficiency in the allocation of public resources, and successive laws and strategic plans for the health service have gradually moved towards establishing the necessary institutions and policies for implementing economic evaluation, progress has been slow and incomplete [31]. The price and reimbursement process in Spain has its particularities [31]. Among them, price and reimbursement decisions have traditionally occurred before the economic evaluation information was considered. Some of the therapeutic positioning reports (IPT), which summarize evidence about the relative efficacy and safety of drugs and must identify the added therapeutic value of a new drug, have recently finally incorporated an economic evaluation.

There is a long tradition in Spain of economic evaluations being produced by research from different initiatives and institutions, such as the Spanish Network of Health Technology Assessment Agencies (RedETS), research academic groups, private consultancies and pharmaceutical companies. Nevertheless, there has been a dearth of formal structures to facilitate the use of economic evaluation to inform health and public health decisions, even though the Spanish constitution states that the allocation of public resources must be equitable and meet the criteria of efficiency in health related decisions [31–33] as well as in other decisions concerning public resources. Having said that, it seems that there is now political will, from the General Directorate of Pharmacy and Health Products at the Ministry of Health, to introduce economic evaluation in the drug related decisions and some steps have been recently taken in this direction [34, 35].

Building on that, in this opinion piece we argue that RedETS and other public health-related institutions should widen and strengthen their capacity to better influence, by means of economic evaluation, the HTA decisions taken by Ministry of Health. In addition, any health-related public institution should be able to understand and become familiar with the incorporation of economic evaluation as a formal tool to consider efficiency on any health-related decisions. There is not a wide culture of generating and producing empirical evidence of how research could affect health outcomes and policy-making. Indeed, there is not sufficient knowledge about the current level of influence of economic evaluations on Spanish health policies. Important work in terms of research impact assessment has been conducted in Catalonia [36–38], with further studies aiming to show how research impact assessment could provide lessons to increase impact [38]. Across Spain, however, current use of research is considered to be too limited, and more extensive knowledge on this is still needed with regard to economic evaluation and efficiency related research [31].

In the above context, this opinion piece aims to promote the case for leaders of the Spanish health, and health research, systems to take further steps that might increase research use in policy-making. To inform this, we present additional perspectives that are broadly consistent with key points from the international literature above, but further strengthen and elaborate it. We do this by providing positive examples of the value both of collaboration, and of a systems approach with a comprehensive and coherent strategy to provide a framework for effective action. First, we describe two recent reviews conducted by authors of the current paper (SH and SP) that strengthen the evidence base on ways to increase the chances of research utilization in policy-making, specifically including economic evaluations. Second, we outline new personal reflections—insider accounts—on two recent Spanish economic evaluations conducted by authors of this paper (MTB and SP) that have informed health policy-making in ways that will improve health.

## Promoting the case that research can be integrated into public health policy-making

### Examples from the international literature on factors enhancing use of research in policy-making

We start the main section by briefly focusing on the two further recent international studies that complement much of the existing literature, but use different types of approaches than those more standard ones used in the studies and reviews discussed in the account of the literature above. By reviewing the relevant global literature from unique perspectives, they distilled some core messages about arrangements that might be likely to encourage research use in health policy-making where it is appropriate and feasible to do so.

The first of these studies [39] examined the findings from two connected reviews of health research impact assessments [40, 41]. From these reviews, the team identified all the assessments of the impact (on policies, practice and improved health) that focused on research conducted through multi-project programmes. The resulting new international dataset consisted of 36 assessments of multi-project programmes. The number of component projects making up each of these 36 programmes ranged from eight to 178. Nine of the 36 programmes consisted entirely, or partly, of HTAs. Thirty-one of the 36 assessments specifically examined the impact on policy made by the projects in the relevant programme (a further three assessments examined impact using a combined policy and clinical category). In these 31 programmes, the median was 35% of projects reporting an impact on policy. In the 25% of programmes with the fewest projects making an impact on policy, only 20% or less of the projects informed policies. At the other end of the spectrum, in each of the 25% of programmes with the most projects making an impact on policy, 70% or more of the projects informed policies [39].

A different approach came in next, because having identified a collection of research programmes where the percentage of individual projects making an impact on policy (as broadly defined) was high, the team then worked backwards to examine the characteristics of those programmes. These programmes generally displayed one or more certain characteristics that sometimes overlapped. These programmes often funded individual projects based on the needs of the relevant healthcare system. The team also reported that “impact is more likely to be achieved when the topics of applied research, and how it might best be conducted, are discussed with potential users of the findings, and when mechanisms are in place to receive and use the findings” [39]. These three characteristics (i.e. based on the needs of the healthcare system, discussed with users, and linked to the creation of structures embedded in policy-making bodies to receive and use the findings from research) are central to the further analysis in this paper. The third one was particularly relevant for the nine HTA programmes for which a median of 77% of projects had a clear impact on policy. The study noted that observations such as the importance of collaboration with users around the needs of the healthcare system, and the importance of the (receptor) bodies receiving the findings, were broadly consistent with the stream of analysis in the United Kingdom and Canada that we reported above in the section on the global literature [22–25].

While some of the three characteristics can be strengthened through good personal relationships between researchers and users—especially at the interfaces—across the board they are stronger if regularized as far as possible, for example in requests for research funding proposals to demonstrate prior stakeholder engagement. Steps could be taken separately to encourage application of each of the three characteristics of priority-setting to reflect healthcare needs, stakeholder engagement and the creation of structures to receive and use research evidence. However, next we turn to the evidence from the second of our two studies which suggests a more concerted approach would be even better.

This second study reported the findings of a WHO-commissioned evidence synthesis of the literature on policies and tools for strengthening national health research systems in order to improve health policies and population health, and contribute to achieving the SDGs [42, 43]. The study found comprehensive strategies were often important in developing research systems that undertook a range of key functions. Many of these were mutually supportive, and included not only the three characteristics noted above, but others that could further boost research use, for example, building research capacity in ways that could conduct, review and disseminate policy-relevant research. Including research utilization as a criterion in research assessment to increase the incentives for researchers to spend time promoting impact is another positive feature of an increasing number of health research strategies. The Catalan Strategic Plan for Health Research and Innovation is a leading example of this in Spain [38].

The WHO evidence synthesis illustrated many of the points by analysing how a comprehensive strategy was important in the success of the National Institute for Health Research (NIHR) created in England in 2006. An overall aim of that strategy was, as stated by its director, “integrating a health research system into the healthcare delivery system so that the two would become interdependent and synergistic” [44]. The *World health report 2013* mirrored such thinking by stating that for maximum effect on policy and practice, “health research should be embedded as a core function in every health system” [1]. Developing the capacity and organizational structures of receptor bodies in order to increase the likelihood of research findings being used might involve actions going well beyond the usual, or perhaps formal, remit of a health research system. Nevertheless, this type of approach is noted as key to the success of the “interfaces and receptor” model. As seen in the United Kingdom, there is likely to be most research impact on policy where there is a dual development of the health research system (integrated into the healthcare system) and receptor bodies in the healthcare system, such as the National Institute for Health and Care Excellence (NICE) and the National Screening Committee. Such receptor bodies were some of the major bodies that had the capacity regularly to receive, analyse and use the findings from projects (including economic evaluations) funded by major NIHR research programmes such as the HTA programme [41, 45, 46].

### Two case studies of the influence of Spanish economic evaluation research on healthcare policies

#### Tobacco control

This Spanish example is best explored in the context of an earlier study in the United Kingdom on which it is built. A research team at Brunel University London worked with a range of stakeholders, including NICE, to develop a Tobacco Control ROI Tool. This helped local authorities and regional tobacco control offices in England devise their policies (and “count the cost of smoking”, according to NICE) because it enabled them to estimate the health and economic returns from possible packages of evidence-based tobacco control interventions [47–49].

The team in the United Kingdom worked with others to develop the EU-funded European-study on Quantifying Utility of Investment in Protection from Tobacco (EQUIPT) project to adapt the decision-support tool to help five countries, including Spain, to make progress with their evidence-based tobacco control programmes. The first author (MTB) was originally part of the United Kingdom team, and then led the Spanish arm of the EQUIPT project [50]. EQUIPT was a comparative effectiveness research project in tobacco control, funded by the European Commission's Seventh Framework Programme. Local policy-makers and public health procurers often lacked the data and financial justification to make the case for tobacco control investments. There was considerable cost-effectiveness evidence for individual interventions within the smoking cessation and tobacco control area, but most of this was deemed insufficient for decision-makers for two reasons. The first was about the evidence base, which had usually been generated from wider contexts, and that did not necessarily resonate with local populations and their needs. The second was about the lack of user-friendly decision-support tools that synthesized costs, effectiveness and other relevant data for a large number of interventions into a single ROI metric. The EQUIPT project addressed these two gaps.

Frequent engagement with a range of relevant stakeholders in each of the participating countries had always been a key aim of the EQUIPT project, as it had been in the original project in the United Kingdom [48]. This was partly to enhance the likelihood that the project would meet the needs of the local healthcare system and population. Such engagement was an important element of the EQUIPT project in Spain [50], where the project’s results were considered in 2019 to inform the policy decision whether to publicly fund, for first time, a pharmaceutical smoking cessation treatment. The president of the National Conference of Tobacco Prevention confirmed that, from its inception, the project engaged with Spanish stakeholders and helped convince the government to finance smoking cessation medications [personal communications with Trapero-Bertran, letter of support 22nd January 2021]*.*

Principal stakeholders from the Spanish Ministry of Health who were in charge of preparing the needed documentation to inform decisions, directly contacted an author of the Spanish publication to request an update on the different prices and costs of the pharmacological treatments in order to update the EQUIPT study results. Updated results were calculated and handed to the Ministry of Health as a confidential report. The Director of Public Health in the Ministry confirmed that on this occasion the results from EQUIPT’s cost–utility analysis influenced the decision to a fund specific type of pharmaceutical smoking cessation treatments [personal communications with Trapero-Bertran, letter of support 27th January 2021]*.*

#### The Cervical Screening Programme

As noted above, the study by Reeves et al. in 2019 reviewed economic evaluations of strategies directed towards enhancing the implementation of public health interventions and policies in high-income countries [8]. It identified 14 empirical studies from 1990 to November 2017. One of these focused on a public health intervention implemented in Spain [51]. That economic evaluation with lead author MTB is the focus of this case study.

The Cervical Screening Programme (CRICERVA) study was initiated in 2011 in the Catalonian public healthcare system where there was an existing opportunistic preventive screening programme, which included the proactive offer of screening at times when women of a relevant age had not yet been screened. Due to the high prevalence of cervical cancer cases, the CRICERVA research team [52] proposed to improve the screening coverage through introducing a population-screening programme. The screening model proposed was centred on the recruitment of women with no previous cytology. It involved performing cytology and the hybrid capture test for human papillomavirus (HPV) to add diagnostic resolution due to the greater sensitivity of the test and the absence of previous screening/cytology in this population, which meant that these women had a greater risk of cervical disease. Systematic screening would facilitate earlier action in detecting premalignant lesions, helping to reduce the incidence of invasive cancer.

The CRICERVA study aimed to identify and inform the best way to implement a population screening policy. Eligible women were assigned to either a control group or one of three intervention groups each receiving a different strategy aimed at increasing screening coverage through a personalized invitation. These three were (i) a personalized invitation letter; (ii) a personalized invitation letter + an informative leaflet; (iii) a personalized invitation letter + an informative leaflet + a personalized phone call [53, 54]. There was some early interaction between the local primary care team, who had identified the need jointly with regional decision-makers, and researchers such as MTB with expertise in economic evaluation. They aimed to conduct a cost-effectiveness analysis of the three different interventions to promote the uptake of screening for cervical cancer in general practice. All three intervention strategies significantly increased participation in screening compared to the control group [53], and were more cost-effective than opportunistic screening, but the first intervention (i.e. just a personalized invitation letter) was the most cost-effective approach [51].

When the study was underway, two key institutions heard about it, showed their interest and decided they would like to be involved: the Catalan Institute of Oncology (ICO) [55] and the Network Biomedical Research Centre (CIBER) of Epidemiology and Public Health [56]. The ICO’s mission is to work to reduce the impact of cancer in Catalonia. It is a public institute focused entirely on cancer, and addresses the disease in a comprehensive way since it brings together prevention, care, specialized training and research within the same organization. It is a multicentre organization with four university hospitals and another 20 hospitals, and currently is the reference cancer centre for about 45% of the adult population in Catalonia. The CIBER is a centre of excellence in epidemiological investigation, in which professionals from the academic field, public administrations and research centres collaborate to provide updated information on the local health situation, including the major biological, environmental and social determinants involved in the most frequent diseases and the study of inequalities.

The participation and incorporation of stakeholders from these two institutions in the research group was important. There was clear multidisciplinary and collaborative work among the research project investigators and stakeholders of the cancer programme that converted the opportunistic screening programme of cervical cancer to the population-screening programme. Indeed, this research project, born in the primary care setting, encouraged and supported additional external papers from researchers from the key and others institutions while the project was underway [57, 58].

Trapero-Bertran et al. (2017) concluded that their analysis of the most cost-effective intervention to boost programme-screening “encourages including this intervention, sending an invitation letter, in the national policy on screening to prevent cervical cancer” [51]. According to a senior researcher at the ICO who was involved in the economic evaluation element of the CRICERVA project, the study did help to inform and influence the Spanish Ministry of Health’s decision to recommend population-screening for cervical cancer [Personal communications with Trapero-Bertran]. However, the intervention of sending an invitation letter has been incorporated differently across the nation, and some autonomous communities have implemented it (Basque Country) and others not yet (Catalonia). This is due to the decentralized organization of the National Health System (NHS) in Spain.

## Lessons from the international literature and local case studies on integrating research into public health policy-making in Spain

In the case studies, the projects focused on issues of healthcare concern and involved considerable stakeholder engagement at various times. In these ways they illustrated key characteristics identified from the global literature as being associated with research that was more likely to make an impact on policy. These two Spanish examples reinforce some of the lessons from the global literature, and could, therefore, help demonstrate to the relevant authorities in Spain the potential usefulness of promoting a health research system in which the characteristics associated with achieving policy impacts regularly featured in projects. These characteristics include creating an appropriate priority-setting mechanism, featuring policy impact in the criteria for evaluating research, requiring proposals to include commitments to stakeholder engagement, and building mechanisms to receive and use research findings produced by studies targeted at meeting the needs of the healthcare system. Many of these features can be brought together in the interfaces and receptor model.

We recognize that the role played by the research leader, being motivated by the public health interest, was key in the tobacco control case study in order to facilitate the research impact on policy, and thus illustrates some of the complexities of the arguments. In this case the project was based on healthcare needs and constructed to involve extensive stakeholder engagement. Furthermore, the impact on policy also highlighted aspects featured in the literature of the committed researcher actively trying to improve health by working at the research/policy interface to promote policy use of the research evidence they had helped to produce [13, 14]. Nevertheless, the evidence from the literature also suggests that the more comprehensive the strategy developed for strengthening the health research system, the more it would be likely to promote elements enhancing the likelihood that research would more regularly be integrated into public health policy-making. These were reasonable conclusions to draw even before the pandemic arose.

Now, however, the current health context created by the pandemic, while complicated, should be an absolute motivation to create and build stronger connections between policy-makers and scientific evidence. The health crisis is linked to an economic crisis with no historical precedents, implying that going forward, decisions on how to allocate the health resources will need to be taken very carefully and on a well-informed basis. While the nature and topics of the cost-effectiveness research described in the case studies may not be relevant for addressing the immediate COVID crisis, increasing the cost-effectiveness of the healthcare system, and the value or ROI of public health interventions, will become even more important in the aftermath of the pandemic.

Furthermore, by reinforcing some of the lessons from the global literature, the examples would have some relevance for boosting the evidence supporting research use in policy-making in most systems. As noted, however, the specific context is usually important, both in terms of the national situation and sometimes, as here, in terms of the type of research. Therefore, this opinion piece now focuses on Spain, and, in particular, research in fields such as HTA and economic evaluation. It is also the case that, as for many other European countries, EU research funding in Spain is important [42], and, as noted in the introduction, the current Horizon Europe framework highlights HTA and the need for cost-effective and fiscally sustainable health policies.

The main problem in Spain is neither building economic evaluation capacity nor producing research, but rather using it and connecting the research and academic world with the policy-making decisions. Some potential actions or options to build a system in which economic evaluations would more regularly be used in policy-making, and thus integrate economic evaluation into the decision-making in the Spanish NHS, could include some of the following recommendations:

(i) Encourage coordination through creating technical policy-independent structures such as networks between different organizations that produce and use economic evaluation evidence, including academic research groups, public health agencies, and groups and societies with interest and knowledge in evaluation (e.g. Genesis). This would be in order to reduce any duplication of efforts, and increase the focus on priority-setting to meet the needs of the healthcare system.
(ii) Build on work conducted in Catalonia [36–38], and use it to stimulate the use of impact assessments of how economic evaluations contribute to the health policy-making processes, in order to increase the transparency of the processes and learn important lessons for planning reforms.
(iii) Endeavour to use an ROI approach for public money, in a complementary way to economic evaluation, to more directly quantify the monetary returns on public health investment in order to meet the need for greater health system efficiency.
(iv) Create a meeting space for evaluating, from the efficiency perspective, the impact of public health interventions across different sectors or areas, such environment or transport. This would fit with a general awareness of the importance of making progress towards the SDGs as noted in the introduction.

Recommendations (i) and (ii) could be achieved by creating a new structure to collate the information on economic evaluations and establish a common and validated methodology, as some Spanish researchers have been recently advocating [59]. Some changes in methodology have been recently implemented in the Spanish NHS drug evaluation process, where the Ministry of Health seems to be convinced of the value of incorporating economic evaluation [60]. There is, however, still a long way to go to talk about a homogeneous and established methodology for economic evaluation process development and influence on the policy-making process.

In addition, this new structure could lead a better integration of the health systems research originated and conducted by the academic and educational institutions to inform healthcare delivery systems. Some of these connections are informally created through specific individuals but they are not formally established, and their degree of influence on the decision-making process is unknown. For instance, the Spanish Ministry of Health commits some reports and research studies to regional health technology agencies in Spain, such as in the Canary Islands [61–63], that evaluate the efficiency of specific health technologies, but not much information appears to be available about whether these reports influence the decision-making process, or to what degree.

Therefore, the integration of key stakeholders becomes an urgent need in order to meet the interests of policy-makers and the academic research agenda. It is time to set an evaluative culture in Spain in order to be able to take better-informed public health decisions. It puts a duty on the research and academic field to support the informational needs of policy-makers, who, in turn, have the task of integrating this health research evidence and information into the decision-making process.

We recognize that desirable aims such as these have been promoted on various previous occasions in a range of systems, but have often made much less progress than hoped because the barriers faced are always considerable [4–13]. Even in the review by team members that found in some programmes a high proportion of projects that made an impact on policy, the median was 35% of projects reporting a policy impact, meaning that the majority of projects in the majority of the programmes were not able to overcome the barriers to making an impact on policy [39].

Nevertheless, there are perhaps three reasons for hoping there might be more progress this time. First, as the global literature and case studies described here show, there is greater accumulation of knowledge and experience that has now been collated and can be drawn upon. For example, in early 2022 a paper described a major research study that went much further than we have been able to do in an opinion paper, and applied the lessons from the global literature on the use of evidence to inform health policy-making to the circumstances of a particular country, Iran, in order to develop a roadmap for enhancing such evidence use in Iran [64]. We hope that our opinion paper will encourage and help facilitate a more thorough analysis of how to enhance the use of economic analysis in Spanish health policies. Similarly, another new paper has gone further than before in exploring ways to institutionalize evidence-informed policy-making [65]. Second, the pandemic created circumstances in which there has been some rapid integration of evidence into policies in order to save lives [66–68], and expectations that some of this will continue in the future. Third, and overlapping with the second point, the economic strains created by the pandemic increase the need for economic evaluations, and there might be another few occasions in the future where the need would be so imperative; therefore, it would be better to start working now towards a direction along the lines set out above.

## Conclusions

While researchers are often frustrated at the perceived lack of attention to scientific evidence during policy-making processes, and there is an increasingly large literature acknowledging inevitable barriers to research use, there are also a growing number of attempts to identify facilitators encouraging such use. The authors of this opinion piece provide further examples from their own studies of how and why research has been integrated into policy-making. They highlight two recent international reviews to show that characteristics of projects more likely to influence policy-making include a focus on healthcare system needs, use of stakeholder engagement, and research that is conducted for organizations supported by structures to receive and use evidence Such characteristics are likely to occur as part of a comprehensive national health research system strategy, especially one integrated into the healthcare system. Local context is important, so we also describe two policy-informing economic evaluations conducted in Spain that focused on issues of healthcare concern and involved considerable stakeholder engagement.

The literature and examples demonstrate to authorities in Spain the value of developing comprehensive approaches to integrate coordination of the efforts of various research producers and users in the health and research systems, with the aim of boosting needs assessment and stakeholder engagement. Increased assessment of research impact on policy would increase transparency. It would also inform further development of recent positive steps in the organization of the use of economic evaluations in health policy-making in Spain and its autonomous regions.

## Data Availability

Not applicable for this opinion paper.

## Abbreviations

CIBER: Network Biomedical Research Centre
CRICERVA: Cervical Screening Programme
EQUIPT: European-study on Quantifying Utility of Investment in Protection from Tobacco
EU: European Union
HPV: Human papillomavirus
HTA: Health technology assessment
ICO: Catalan Institute of Oncology
LMICs: Low- and middle-income countries
NHS: National Health System (of Spain)
NICE: National Institute for Health and Care Excellence (England)
NIHR: National Institute for Health Research (England)
RedETS: Spanish Network of Health Technology Assessment Agencies
ROI: Return on investment
SDG: Sustainable Development Goal
